# Enhancing the utility of polygenic scores in Alzheimer’s disease through systematic curation and annotation

**DOI:** 10.3389/fgene.2025.1507395

**Published:** 2025-02-04

**Authors:** Savannah Mwesigwa, Yulin Dai, Nitesh Enduru, Zhongming Zhao

**Affiliations:** ^1^ Center for Precision Health, McWilliams School of Biomedical Informatics, The University of Texas Health Science Center at Houston, Houston, TX, United States; ^2^ Faillace Department of Psychiatry and Behavioral Sciences, McGovern Medical School, The University of Texas Health Science Center at Houston, Houston, TX, United States

**Keywords:** Alzheimer’s disease, genetic variant, PGS catalog, polygenic scores, rank aggregation

## Abstract

**Introduction:**

Polygenic Scores (PGSs) assess cumulative genetic risk variants that contribute to the association with complex diseases like Alzheimer’s Disease (AD). The PGS Catalog is a valuable repository of PGSs of various complex diseases, but it lacks standardized annotations and harmonization, making the information difficult to integrate for a specific disease.

**Methods:**

In this study, we curated 44 PGS datasets for AD from the PGS Catalog, categorized them into five methodological groups, and annotated 813,257 variants to nearby genes. We aligned the scores based on the “GWAS significant variants” (GWAS-SV) method with the GWAS Catalog and flagged redundant files and those with a “limited scope” due to insufficient external GWAS support. Using rank aggregation (RA), we prioritized consistently important variants and provided an R package, “PgsRankRnnotatR,” to automate this process.

**Results:**

Of the six RA methods evaluated, “Dowdall” method was the most robust. Our refined dataset, enhanced by multiple RA options, is a valuable resource for AD researchers selecting PGSs or exploring AD-related genetic variants.

**Discussion:**

Our approach offers a framework for curating, harmonizing, and prioritizing PGS datasets, improving their usability for AD research. By integrating multiple RA methods and automating the process, we provide a flexible tool that enhances PGS selection and genetic variant exploration. This framework can be extended to other complex diseases or traits, facilitating broader applications in genetic risk assessment.

## Introduction

Alzheimer’s Disease (AD) is a neurodegenerative disorder characterized by gradual cognitive decline and memory loss. Genetic predisposition plays a significant role in AD etiology, with numerous susceptibility loci identified through genome-wide association studies (GWASs). Heritability estimates for AD, which range from 60% to 80% (Bergem et al., 1997; Gatz et al., 2006), underscore the importance of genetic factors in understanding AD risk.

The Polygenic score (PGS) is a numerical estimate of an individual’s genetic susceptibility to a trait or condition, computed by summing the effects of the individual’s genome-wide genotypes, weighted by effect size estimates derived from genome-wide association studies (GWAS) summary statistics (Choi et al., 2020). Therefore, Polygenic risk scores (PGSs) offer a promising approach to integrating multiple genetic variants and estimating an individual’s genetic susceptibility to AD (Leonenko et al., 2019). With the rising popularity of PGSs in recent years, the PGS Catalog (https://www.pgscatalog.org) was developed (Lambert et al., 2021) to tackle the lack of established best practices and reporting standards that pose a significant barrier to PGS research. The PGS Catalog is an open resource that offers a comprehensive compilation of published PGSs, along with detailed metadata. This metadata includes variant scoring files, effect alleles/weights, and predictive performance evaluations, ensuring the accurate application and evaluation of PGSs.

Researchers have employed various methodologies to generate PGSs that capture the genetic risk associated with the AD; GWAS Significant Variants (GWAS-SV) select top hits from genome-wide association studies; LASSO uses regression-based selection and regularization to handle large datasets with many predictors (Privé et al., 2020a; Mak et al., 2017); Bayesian approaches such as DBSLMM, LDpred2, PRS-CS, and SBayesR (Yang and Zhou, 2020; Privé et al., 2020b; Ge et al., 2019; Lloyd-Jones et al., 2019) incorporate prior knowledge and probabilistic modeling to account for uncertainty and LD; and ensemble methods which combine multiple models/summary statistics to improve predictive performance by leveraging the strengths of each (Zhang et al., 2021; Sofer et al., 2023).

However, the inherent variability in method and number of variants in the PGS catalog hinders researchers from easily identifying appropriate scores for reuse due to variations in reliability and accuracy based on the methodologies and quality of the GWAS used. Recent efforts have been made to significantly improve the reporting standards for PGSs. Wand et al. (Wand et al., 2021) outlined guidelines to enhance the reproducibility and standardization of PGSs, while Lambert et al. (2024) expanded the PGS Catalog, improving data content and interface to support FAIR principles (Findable, Accessible, Interoperable, Reusable) (Wilkinson et al., 2016; Xiang et al., 2024), thereby facilitating reproducible research and equitable application across diverse populations.

To achieve these goals, we aim to bridge this gap by developing a unified curation standard with harmonized annotations, simplifying the exploration of diverse PGSs and their associated genetic variants and genes. We used AD as a demonstration trait to curate and annotate a database of PGSs from the PGS catalog, where we manually classified methodologies and annotated the variants based on detailed examination of metadata and associated documentation, to ensure accurate classification of PGS datasets. Variant annotations were conducted through systematic mapping to nearby genes by using established genomic resources such as the University of California Santa Cruz (UCSC) Genome Browser database. Additionally, we provided multiple rank aggregation (RA) options to consolidate variant rankings based on effect weights, providing researchers with a streamlined method to quickly explore variants that consistently have high effect weights across multiple PGSs. Finally, we deployed a publicly available R wrapper package, “PgsRankRnnotatR,” to accelerate the variant annotation and RA processes.

## Methods

### Curation of PGSs for AD

We downloaded AD-related PGSs from the PGS Catalog (accessed in December 2023) and harmonized variant positions to the GRCh38 human reference genome build. Utilizing the Quincunx package in R (Magno et al., 2022), we queried the catalog’s REST API with the trait term ‘Alzheimer’, extracting a total of 44 PGSs. We applied the R package ‘dplyr’ (Wickham et al., 2023) to aggregate these PGSs into a singular cohesive dataset to facilitate downstream analyses.

### Variant annotation and methodological classification of PGSs

We used the R package “annotatr” (version 1.20.0) to annotate variants with the nearest gene using the UCSC annotation databases. Gene annotations were primarily based on proximity due its simplicity, though it has inherent limitations in capturing intergenic variants. Quality checks were conducted to ensure the standardization of allele nomenclature (rsIDs), filtering out non-standard variant labels like APOE ε2, ε3, and ε4, which were excluded because the three labels are defined by a combination of two SNPs, rs429358 and rs7412, which cannot be easily integrated into the single-SNP weighting framework of this study. To maintain consistency, only standard SNP identifiers were considered. We classified PGSs by methodology into five main categories: “GWAS significant variants (GWAS-SV)”, “Clumping and thresholding”, ‘Bayesian’, ‘Least Absolute Shrinkage and Selection Operator (LASSO)”, and “Others”. For PGSs using the GWAS-SV, redundant files were identified as those developed from the same original GWAS, and therefore utilized identical variants and weights; this redundancy was confirmed through systematic review of bibliographic metadata and variant annotations. When additional variants were provided by authors, a single representative file was retained, prioritizing those closely aligned with the original GWAS variants to ensure consistency.

### Rank aggregation of genetic variants

We implemented a RA framework to prioritize genetic variants with consistent relevance across multiple PGS datasets. The ranking process was based on the absolute values of PGS effect weights, reflecting each variant’s relative contribution within individual PGSs, while GWAS effect sizes (odds ratios) from the GWAS Catalog were used to validate and contextualize the aggregated rankings.

To benchmark six different RA techniques, we generated consensus rankings of the variants based on their effect weights. Among them, the Dowdall method was implemented via our custom R script to calculate the mean of the reciprocals of variant ranks, where ranks range from 1 (highest rank) to N (lowest rank, where N represents the total number of variants) (Grofman et al., 2017). For multiple queries (score files) Q, the mean reciprocal rank (MRR) score is the mean of the Q reciprocal ranks. MRR scores values range from 0 to 1, where the score closer to 1 indicates consistently high rank, while the score closer to 0 indicate lower rank. Variants were then re-ranked based on their MRR scores, with higher MRR scores resulting in higher aggregated ranks. We generated a rank matrix for all the aggregated PGSs, assigning variants with missing effect weights in a particular PGS the lowest rank, N.

$$\frac{1}{Q}\sum_{i=1}^{Q}\frac{1}{{rank}_{i}}$$

For other methods, we utilized the RobustRankAggreg package (version 1.2.1) in R inputting ranks normalized by Min-Max scaling. We applied five algorithms from the package: Robust Rank Aggregation (RRA) works by comparing the actual ranks to a null model of random orders to identify high rankings consistent across studies and adjusting for multiple testing (Kolde et al., 2012); The Stuart Method aggregates ranks by calculating corrected p-values from a joint cumulative distribution of order statistics and ultimately using these to derive a final aggregated rank that measures the statistical likelihood of observed rankings occurring by chance (Stuart et al., 2003); Borda’s Methods include the Minimum Rank that prioritizes variants based on the highest rank position (meaning the lowest numerical value) across all rankings; the Geometric Mean that calculates the *N*th root of the product of all ranks, where N is the total number of ranks. Lastly, the Mean of Ranks as a straightforward statistical measure for a central tendency of ranks (Wang et al., 2022) (Supplementary Text S1).

Then, we evaluated the performance of these six RA methods using a ‘GWAS priority score,’ presenting the product of the ‘Average effect size’ and the ‘Number of Genome-Wide Significant Associations with AD.’ The ‘Average effect size’ was calculated as the absolute value of the log-transformed odds ratio. We utilized the R package ‘gwasrapidd’ (version 0.99.17) (Magno and Maia, 2020) to retrieve effect sizes and the counts of GWAS significant associations for each genetic variant. Therefore, this score provides a measure of the relative importance of each variant based on its impact and the robustness of its association with AD. We evaluated the different RA methods by comparing how well the aggregated ranks correlated with the GWAS priority scores. The top-ranked PGS variants would demonstrate high GWAS priority scores.

To streamline aggregating, ranking, and annotating the PGS variants, we wrapped the PgsRankRnnotatR into an R package (Supplementary Figure S1).

## Results

### Workflow for curating PGSs

**FIGURE 1:**
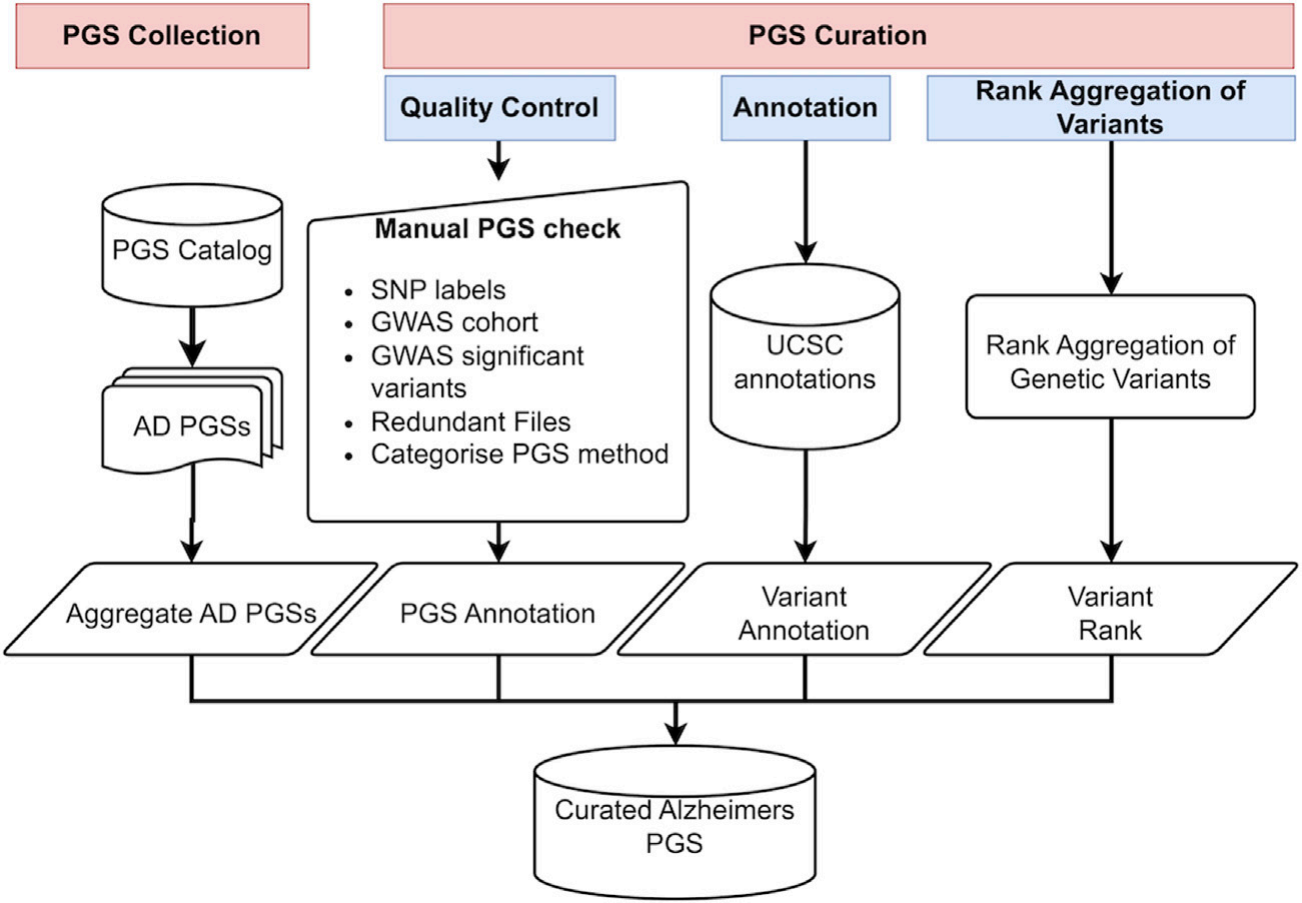
Study design. AD PGS datasets were collected and subjected to quality control steps and manual annotations, genetic variants were annotated and ranked, and then the final RA score was computed.

To address the need for a standardized approach in collecting, annotating, and prioritizing variants from PGSs for a specified trait such as AD, we developed “PgsRankAnnotatr,” an R-based wrapper package to automate the acquisition, annotation, and RA of PGS variants for specific trait using data from the PGS Catalog. Figure 1 illustrates an overview of the workflow for curating and annotating PGSs, which consists of two main steps: retrieving PGSs from the PGS Catalog and conducting PGS curation. The PGS curation process encompasses three main functions: quality control, variants annotation, and variants rank aggregation.

### AD PGS studies curation

Following the workflow outlined in Figure 1, we retrieved 44 AD PGSs from the PGS catalog (see Supplementary Table S1 and Supplementary data for details). We observed significant variability in the number of variants used to construct PGSs for AD, ranging from six (Tanigawa et al., 2022) to over one million variants (Monti et al., 2023). This variation reflects methodological differences, with GWAS-SV methods prioritizing fewer, highly associated variants for better interpretability, and Bayesian approaches (Yang and Zhou, 2020; Privé et al., 2020b; Ge et al., 2019; Lloyd-Jones et al., 2019) integrating a broader spectrum of genetic data for potentially improved risk assessment at the cost of higher computational demands and underscores the diverse utilities and purposes of AD-related PGSs.

**FIGURE 2:**
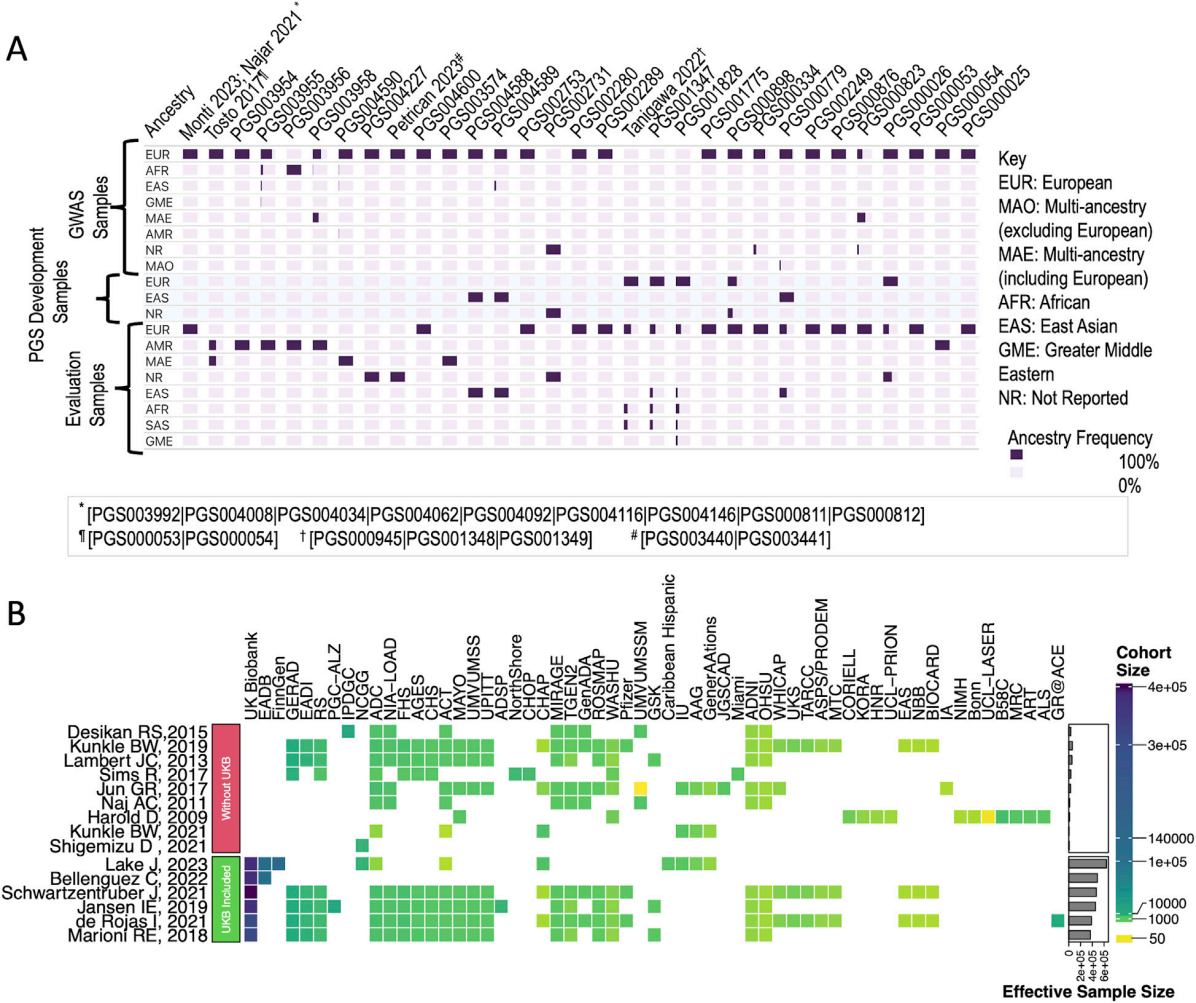
Overview of AD PGSs curation **(A)** Ancestry group frequencies of cohorts used for PGS development and evaluation. Each cell represents the frequency of a specific ethnic group (row-wise) for various PGSs (column-wise). While most PGSs were developed using predominantly European ancestry cohorts, 14 out of 27 PGSs were evaluated in more diverse non-European cohorts. Scores with identical ethnic group frequencies are grouped under a single citation (author, year) with details in the figure legend. **(B)** Heat map of discovery cohort sizes in GWAS for polygenic score variant selection. A visual representation of the discovery cohort sizes of the GWAS was used to select variants for PGS development. Each row corresponds to a different GWAS or meta-analysis, while each column represents a specific cohort used for data generation. One cluster predominantly consisted of studies leveraging the UK Biobank cohort to increase sample sizes (Green box) substantially. The other cluster primarily comprised older studies that did not utilize the UK Biobank cohort (Red box).

The variants utilized for PGS development primarily originate from GWAS conducted on cohorts of European ancestry (Figure 2A), drawing from data from 15 GWASs (Figure 2B), which also depicts the need for a broader spectrum of GWAS focusing on AD with diverse ancestral backgrounds. Notably, certain large AD GWAS, such as the one conducted by Wightman et al. (2021), were absent from the datasets utilized for PGS development. However, the cohorts used for evaluating the PGSs exhibited greater diversity, with 14 PGSs evaluated in non-European cohorts (Figure 2A). Four PGSs from two studies (Privé et al., 2022; Tanigawa et al., 2022) relied on individual-level genetic and phenotypic data from the UK Biobank rather than GWAS summary statistics. Upon cross-referencing with the GWAS catalog, it was observed that the variants included in these scores lacked external validation through independent GWAS datasets. Although this does not compromise the methodological quality or validity of these scores, the absence of external validation limited their compatibility with the harmonization framework used in this study.

### Various PGS methods utilize different numbers of genetic variants

**FIGURE 3:**
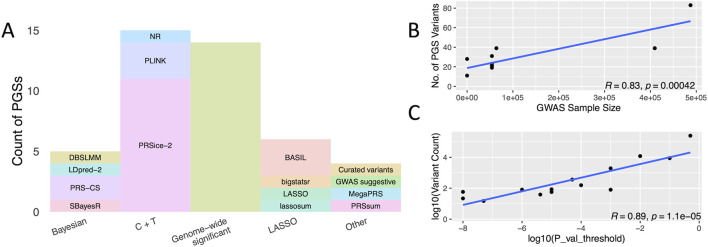
**(A)** Methods for the polygenic risk score calculation. The stacked bar plot depicts the prevalence of different tools classified by polygenic risk score (PGS) calculation methodology. Methods utilizing GWAS-SV and C + T dominate the landscape, particularly in AD studies, followed by LASSO regression, Bayesian methods, and alternative or ensemble approaches. Direct Correlation between PGS variants and GWAS characteristics. **(B)** The plot demonstrates a direct proportionality between the number of genome-wide significant variants used to compute the PGS and the GWAS sample size. **(C)** The plot illustrates a similar direct relationship between the number of variants in the PGS and the GWAS p-value threshold.

We found that the Clumping and Thresholding (C + T) method (Euesden et al., 2015) was the most frequently used for PGS generation, appearing in approximately 34% of the analyzed studies, as depicted in Figure 3A. This method is widely used for its computational efficiency while accounting for the impact of linkage disequilibrium (LD) between SNPs. It enables the selection of independent genetic variants that significantly contribute to AD risk. Subsequently, the use of GWAS-SV constitutes 32% of the approaches. These methods are preferred for their direct focus on variants with robust statistical evidence linking them to AD. However, we identified one file containing variants not represented in the GWAS catalog, despite being labeled as GWAS-SV, and excluded it from our RA analysis.

Our systematic review identified ten redundant files originating from four GWASs, as outlined in Supplementary Data. To ensure unbiased RA analyses and avoid overrepresentation of variants, these redundant PGSs were excluded from the dataset. The LASSO and Bayesian methods were used less frequently in 14% and 11% of the studies, respectively. LASSO is favored for its ability to handle large sets of predictors by simultaneously performing variable selection and regularization, which can be helpful for complex traits like AD.

Bayesian methods require intensive computational resources and more sophisticated analysis, possibly explaining their lower prevalence. These findings underscore the diversity of methodologies in generating PGS for AD, indicating that preferences are influenced by a balance among computational demands, analytical precision, and specific research goals. In addition to the primary methodologies identified, we identified a subset of approaches we classified under ‘Other,’ accounting for approximately 9% of the methods utilized, as illustrated in Figure 3A. This category encompasses strategies such as curated variants, selected based on expert knowledge or specific criteria beyond statistical significance, and ensemble methods, which combine predictions from multiple PGS generation techniques to increase the accuracy and robustness of the risk scores (Zhang et al., 2021; Sofer et al., 2023). Incorporating curated variants signifies a customized approach to PGS development, prioritizing genetic markers with established biological relevance to AD. Conversely, ensemble methods aim to harness the advantages of diverse predictive models to mitigate their individual constraints. Though less prevalent, these alternative approaches demonstrate continuous exploration and adaptation in PGS research.

### Influence of GWAS on PGS selection

Larger GWASs have greater statistical power to discover more genome-wide significant variants, leading to a direct correlation between the GWAS sample size and the number of variants included in PGS methodologies using GWAS-SV, as depicted in Figure 3B. On average, this approach utilized the fewest variants compared to others (Table 1). In C + T methods, variant selection heavily depends on the p-value threshold set by researchers, resulting in a wide range of variant numbers, as shown in Figure 3C. Notably, the ‘Bayesian’ methods incorporated the most extensive set of variants, as highlighted in Table 1.

**TABLE 1:** Summary of PGS methods by category and the variant count.

Method category	No. of variants		
	Min	Median	Max
Bayesian method	915,771	1,092,011	1,136,212
Clumping and Thresholding	15	85	249,273
Genome-wide significant variants	11	26	83
LASSO	6	15	5,663

Conversely, we identified four PGS files derived from LASSO, which were categorized as having a ‘limited scope’ and thus omitted from our RA analysis. We used the term “limited scope” to describe the restricted applicability of these scores due to the lack of validation of the variants through external GWAS data. While the individual-level data they utilize is valuable, external GWAS validation ensures broader applicability and reliability across different datasets and populations. These PGSs, developed independently of GWAS and relying solely on individual-level genetic and phenotypic data, lacked GWAS validation for the selected variants. This highlights the critical role of external GWAS validation in affirming the reliability and relevance of PGSs for AD research.

### Comparison of ranking algorithms

**FIGURE 4:**
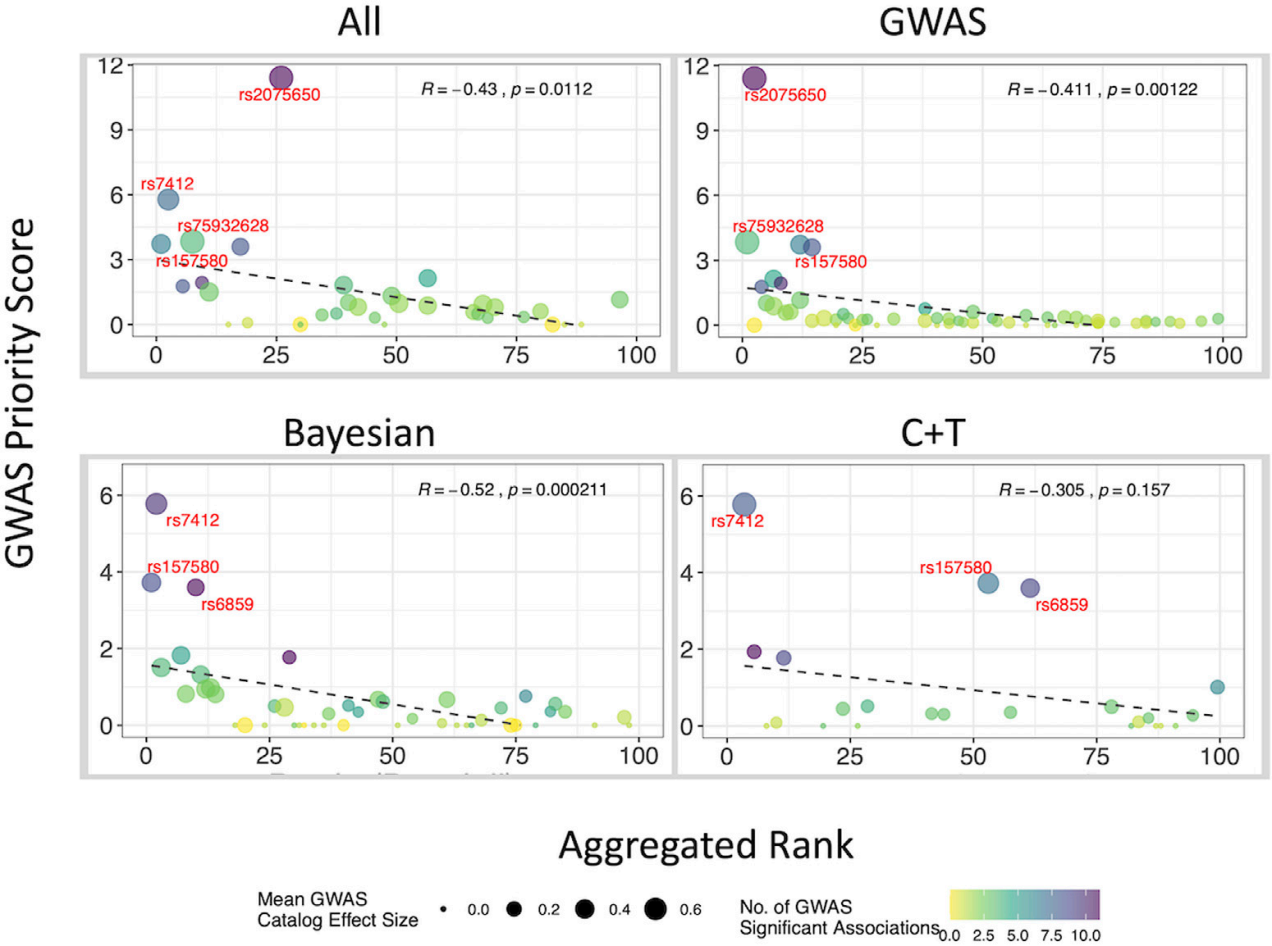
A scatter plot for visualizing the variants by rank aggregation (RA) score (Dowdall method) and GWAS priority score. This scatter plot shows the negative correlation between the top 100 AD genetic variants based on the Dowdall RA score and the GWAS priority score. Variants were categorized by methodology type before performing RA analysis. Due to clumping, the C + T method had fewer GWAS Catalog variants than the Bayesian or GWAS-SV method. Top GWAS priority score variants are labeled in red.

We evaluated six RA methods based on their Pearson’s correlation coefficient with the GWAS priority score across four datasets: the combined dataset (“All”), “GWAS”, “Bayesian”, and “C + T” (Supplementary Figure S2). As summarized in Supplementary Figure S2, the average correlation coefficients (R_mean_) were: Geometric Mean (R_mean_ = −0.39), Dowdall (R_mean_ = −0.42), Stuart (R_mean_ = −0.38), Minimum (R_mean_ = −0.42), Robust Rank Aggregation (R_mean_ = −0.17), and Mean (R_mean_ = −0.30). The Dowdall and Minimum rank methods showed the strongest correlations, but Dowdall offered a key advantage through its continuous scoring system (Supplementary Figure S2). With the Minimum rank, multiple variants can share the same aggregated rank; however, the Dowdall provided unique scores that allow for finer differentiation, particularly in the combined (“All”) and “C + T″ datasets. The GWAS priority scores for AD variants ranged from 0 to 27.19 (rs429358), with a median value of 0.04 and a mean value of 0.39. Most variants had low or no priority scores, while a few showed significantly high values, reflecting their strong association with AD. Through RA, we can visualize how different PGS development methodologies impact the variants used. For example, the C + T method had fewer GWAS catalog variants due to clumping compared to GWAS-SV and Bayesian methods (Figure 4). Furthermore, we used the “PgsRankAnnotatR” tool to curate and rank variants for additional traits, schizophrenia and cognition (Supplementary Figure S3), and integrated these into an AI-driven chatbot framework, GENEVIC (GENetic data Exploration and Visualization via Intelligent interactive Console) (Nath et al., 2024), which provides an interactive conversational interface for querying and exploring ranked PGS variants using the Dowdall method. This demonstrates the practical utility of our resource in real-world research workflows.

## Discussion

PGSs have been recognized as crucial tools for assessing individual’s genetic risk in AD (Escott-Price et al., 2015), with diverse methodologies and GWASs contributing to an extensive range of available scores (Zhou et al., 2021). This flexibility, while contributing to methodological heterogeneity, also reflects efforts to enhance the accuracy, robustness, and biological relevance of genetic risk assessments for AD. For example, Bayesian methods not only incorporated the most extensive set of variants, offering a comprehensive genetic landscape for analysis, but they also often achieve the highest predictive accuracy for AD risk (Ni et al., 2021). Despite their superior performance, these methods are computationally intensive and pose challenges for clinical application, highlighting the trade-offs between analytical precision and resource requirements (Ni et al., 2021). However, the heterogeneity of these scores poses challenges for researchers aiming to reuse them effectively, and thus, there is a need for systematically curating and annotating the scores. Leveraging the PGS catalog as a core resource, our curated database aims to enhance accessibility and usability for researchers by streamlining the process of reusing these scores. This will not only facilitate the advancements in personalized AD risk assessments but will also bolster the development of targeted interventions, driving forward the broader mission of advancing precision medicine in addressing the disease.

Each method offers distinct advantages depending on the research context and data availability. The curation process involved broadly categorizing methodologies, identifying overlapping scores, and uniformly annotating and ranking the variants. In addition, we provide a rank score that allows for the quick prioritization and exploration of the most impactful genetic variants, based on their consistent significance across multiple PGSs. Furthermore, we developed an R package, ‘PgsRankRnnotatR,’ as a supplementary tool to automate this process. This allows researchers to select scores that best align with their genotyping resources, ranging from basic GWAS-SV to more comprehensive PGSs. Additionally, incorporating RA methods for identifying ‘priority variants’ should enhance the database’s utility, enabling a more focused exploration of genetic variants.

Although our curated database is based on the PGS Catalog, and there are other AD genetic databases, such as the Alzheimer’s Disease Variants Portal (ADVP) (Kuksa et al., 2022) and similar resources that compile genetic association findings from the literature, our database stands out by offering a comprehensive integration of AD PGSs, along with aggregated variant ranks to facilitate the exploration of risk variants.

Our current curated database still has several limitations. First, the prioritization of variants through RA, though adequate for highlighting consensus genetic variants, may overlook rare but potentially influential variants. Additionally, our ‘GWAS priority score’ is limited to GWAS significant variants reported in the GWAS catalog with odds ratio effect sizes, which may exclude potentially relevant variants. We recognize that annotating variants based solely on proximity may not fully account for intergenic variants, which represent a substantial portion of GWAS findings. Future versions of this resource could incorporate additional functional datasets, such as various QTL annotations [e.g., expression QTL (eQTL), methylation QTL (mQTL), protein QTL (pQTL)], available from general resources such as GTEx (gtexportal.org) and the resources for specific diseases such as FunGen-AD (https://adsp-fgc.niagads.org/). This would enhance the biological relevance of the annotations and broaden the tool’s applicability. There is also the selective exclusion of significant variants such as rs429358 and rs7412 or other variants in the APOE locus in the AD PGS studies given their dominant effect; this is typically justified to aid the discovery of novel variants outside this well-known locus; however, this can also bias the results of RA, potentially underrepresenting the crucial roles of these variants in AD. For example, rs429358, a well-known variant impacting CSF amyloid-β42 levels and dementia risk (Bennet et al., 2010), was included in only 6 out of 44 polygenic scores, and rs7412 was included in 10, resulting in rs7412 having a much higher aggregated rank than rs429358. Variability in variant selection strategies, including the C + T methods, further complicates consistent variant usage across studies. While RA algorithms, such as Dowdall and Minimum rank, demonstrated robustness when handling variants with varying usage across studies, future versions of the ranking procedure could include imputation-based methods to account for missing but biologically relevant variants. Secondly, the database’s breadth is also limited by the overlapping cohorts in the GWAS used for PGS development and the subsequent lack of ethnic diversity, potentially affecting its applicability across different demographic groups. Since overlapping cohorts may introduce bias in RA calculation, future work may address this issue by implementing strategies such as re-weighting the rank.

Additionally, although the manual curation of scores may address nomenclature discrepancies in methodologies, it also imposes limitations on scalability and timeliness because of its labor-intensive nature. Finally, while the RA analysis aimed to aggregate PGS Catalog variants to provide an overall ranking for variant exploration and prioritization, it does not account for LD between genetic variants. Although the different PGS methodologies in our analysis already incorporate LD structures, accounting for LD information directly into the RA process could further enhance the robustness of our variant prioritization.

RA serves as a unique form of meta-analysis, emphasizing consistency across studies rather than pooling effect sizes. Unlike traditional meta-analysis, which assesses the overall strength of associations by combining effect sizes, RA highlights variants that consistently rank highly across diverse PGS methodologies, thus offering researchers with a valuable tool for exploring the curated database and identifying consistent and reliable genetic variants for further study.

We exemplify the real-world utility of “PgsRankAnnotatR” by integrating curated and ranked PGSs into GENEVIC (Nath et al., 2024), an AI-based application that enables researchers to interactively explore genetic variants, and automatically link the results to protein-protein interactions, gene set enrichment analysis and literature mining, without additional tool installation or updates. Future iterations of the application will further refine its capabilities, incorporating additional data sources and case-study validations to support complex trait research.

This paper presents a curated and annotated database of PGSs specifically tailored for AD research. Through curation by classification of methodologies and annotation of genetic variants, we have established a resource that significantly streamlines the retrieval and application of PGSs in AD research. The integration of RA techniques has refined the utility of this database, enabling researchers to prioritize variants with increased precision and confidence. Moving forward, we are committed to continuously updating this database and expanding its scope to include additional traits and diseases, thereby advancing the field of precision medicine, and enhancing our ability to tackle complex genetic challenges.

## Data Availability

The annotated and aggregated PGSs can be accessed via https://zenodo.org/records/11088690. The R scripts/wrapper package used in this study can be accessed and downloaded from our GitHub repository at https://github.com/bsml320/PgsRankRnnotatR.

## Abbreviations

AD, Alzheimer’s disease; C + T, Clumping and Thresholding; GWAS, Genome-wide association studies; LASSO, Least Absolute Shrinkage and Selection Operator; PGS, Polygenic Score; RA, Rank Aggregation; RRA, Robust Rank Aggregation.
