# Supplementary material for: Enhancing the utility of polygenic scores in Alzheimer’s disease through systematic curation and annotation
Source: Front Genet. 2025 Feb 4;16:1507395. doi: 10.3389/fgene.2025.1507395 (PMC11832703; doi:10.3389/fgene.2025.1507395)
Supplement: Supplementary file 1 [file DataSheet1.pdf]

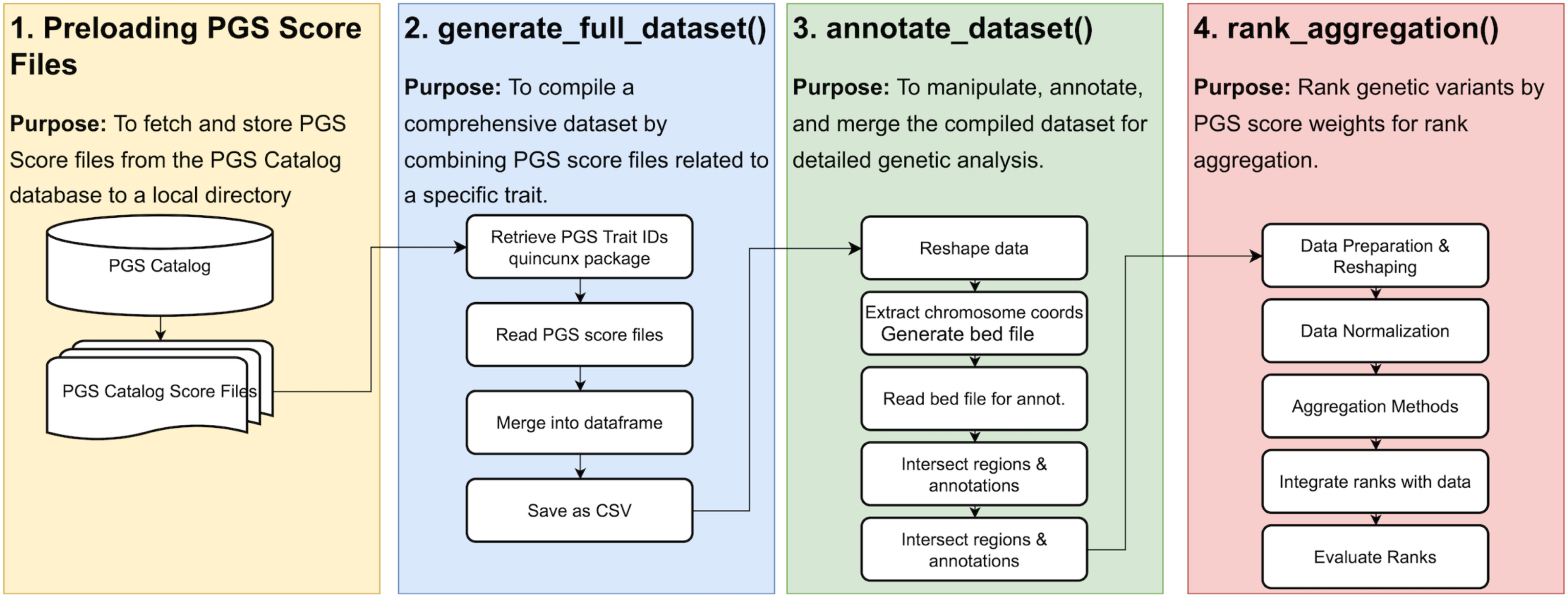

Supplementary Figure 1

Workflow overview of the PGS processing steps using the ‘PgsRankAnnotatR’ R package. The process begins with downloading a local repository of Polygenic Scores (PGSs) from the PGS Catalog (Step 1). Subsequently, three functions of the ‘PgsRankAnnotatR’ package are utilized: The first function aggregates a complete dataset of trait-specific PGSs (Step 2). The second function annotates the variants with nearby gene names and ranks them based on the absolute values of their effect weights (Step 3). The third function performs RA of the variants, optimizing the identification and comparison of significant genetic markers across studies (Step 4).

Supplementary Text S1: Rank Aggregation Techniques

In this study, we benchmarked six rank aggregation (RA) techniques to prioritize genetic variants across multiple Polygenic Score (PGS) datasets. The methods evaluated are summarized below:

1. **Dowdall Method:**
   - This method calculates the mean of the reciprocals of variant ranks across PGS datasets. The formula is: $mean\ reciprocal\ rank = \frac{1}{Q}\sum_{i=1}^{Q}\frac{1}{rank_i}$
   - where Q is the number of PGS datasets, $\frac{1}{rank_i}$ is the rank of the variant in the ith dataset. Variants with consistently high ranks across datasets have higher MRR values. This approach gives more weight to top ranks and is less influenced by outliers at lower ranks.
2. **Robust Rank Aggregation (RRA):**
   - RRA identifies consistently high-ranking items by comparing observed ranks to a null model of random rankings. It adjusts for multiple testing using statistical significance, ensuring robustness to noise and variations across datasets.
3. **Stuart Method:**
   - This technique uses corrected p-values derived from a joint cumulative distribution of order statistics. By aggregating ranks probabilistically, it assesses the likelihood that observed rankings are due to chance. It is sensitive to consistent trends in rank data while controlling for statistical noise.
4. **Borda's Minimum Rank:**
   - This approach prioritizes variants based on their highest rank position (lowest numerical value) across all datasets. It highlights variants that appear in the top positions of any dataset, regardless of their rankings elsewhere.
5. **Borda's Geometric Mean:**
   - This method calculates the geometric mean of the ranks across datasets:
   - $Geometric\ Mean = \left(\prod_{i=1}^{Q} rank_i\right)^{\frac{1}{Q}}$
6. **Mean of Ranks:**
   - This averages the ranks of a variant across all datasets:

$$Mean = \frac{1}{Q}\sum_{i=1}^{Q} rank_i$$

   - It provides a straightforward measure of central tendency but can be sensitive to outliers in the ranking data.

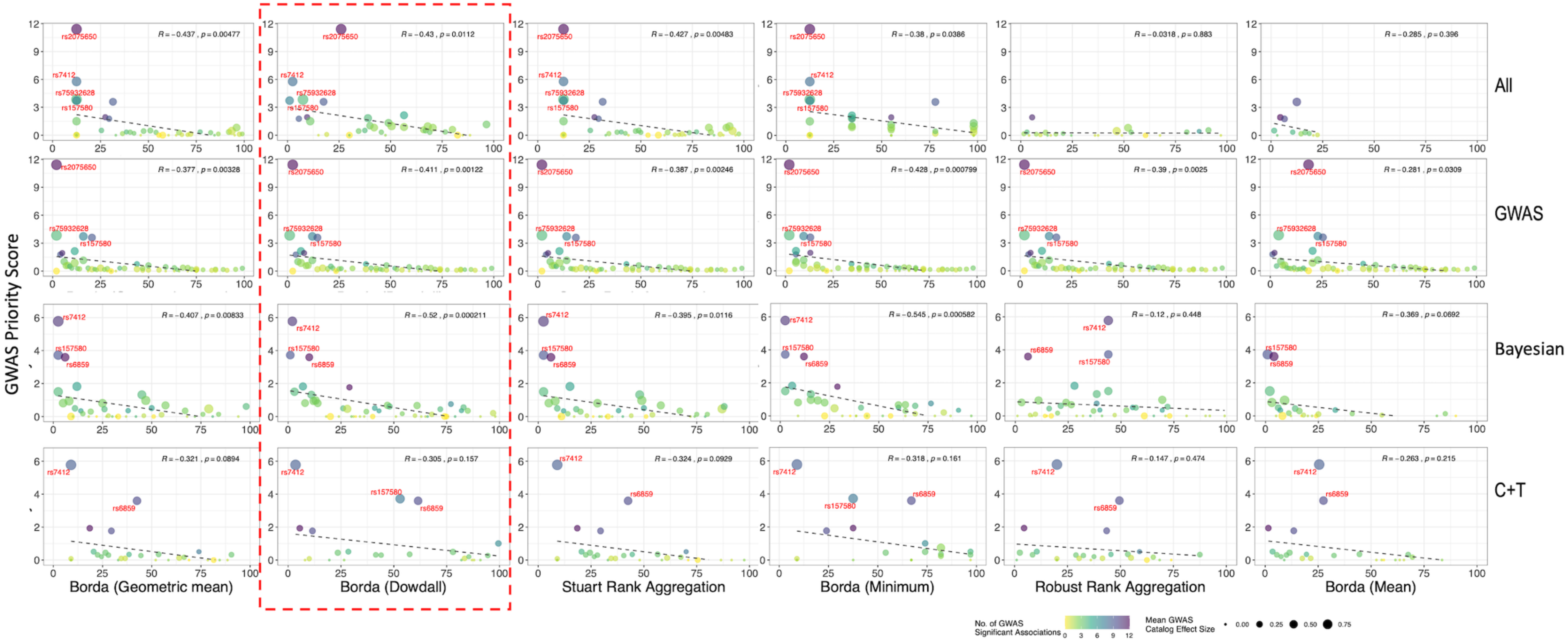

Supplementary Figure 2

Comparison of rank aggregation (RA) methods based on their average Pearson correlation coefficient with the GWAS priority score, computed across four datasets ("All," "GWAS," "Bayesian," and "C+T"). The average correlation coefficients were as follows: Geometric Mean (-0.38550), Dowdall (-0.41650), Stuart (-0.38325), Minimum (-0.41775), Robust Rank Aggregation (-0.17220), and Mean (-0.29950). Both the Dowdall and Minimum rank methods demonstrated similar correlation coefficients, with Dowdall offering the advantage of a continuous scoring system, particularly in datasets like "All" and "C+T" which allows for finer differentiation among variants, where continuous scoring enhances the resolution of rankings compared to the discrete nature of the Minimum rank.

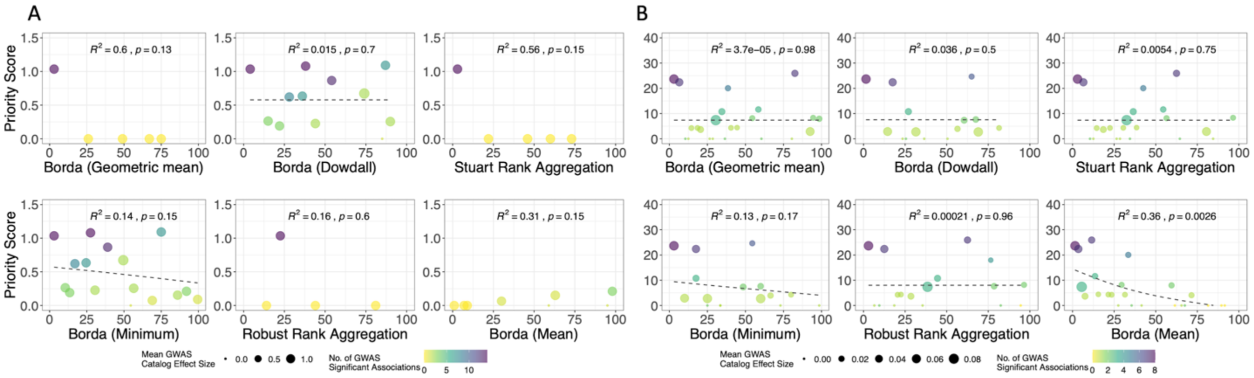

Supplementary Figure 3.

Comparison of rank aggregation (RA) methods based on their average Pearson correlation coefficients with GWAS priority scores, evaluated in a merged PGS dataset for schizophrenia (A) and cognition (B). These datasets were incorporated into the GENEVIC tool [GENetic data Exploration and Visualization via Intelligent interactive Console (PMID: 39115390)] and represent neurological traits with a limited number of Polygenic Score (PGS) files (5 each). Despite the smaller sample size, both the Dowdall and Minimum Rank methods extract top GWAS priority score variants especially in schizophrenia datasets, though their correlation coefficients were not statistically significant.

Supplementary Table 1. References for Alzheimer's disease polygenic scores

PolygenicScoreID	Author
PGS000025	Chouraki V et al. J Alzheimers Dis (2016)[1]
PGS000026	Desikan RS et al. PLoS Med (2017)[2]
PGS000053	Tosto G et al. Neurology (2017)[3]
PGS000054	Tosto G et al. Neurology (2017)[3]
PGS000334	Zhang Q et al. Nat Commun (2020)[4]
PGS000779	Zhou X et al. Alzheimers Dement (Amst) (2020)[5]
PGS000811	Najar J et al. Alzheimers Dement (Amst) (2021)[6]
PGS000812	Najar J et al. Alzheimers Dement (Amst) (2021)[6]
PGS000823	van der Lee SJ et al. Lancet Neurol (2018)[7]
PGS000876	Leonenko G et al. Ann Clin Transl Neurol (2019)[8]
PGS000898	de Rojas I et al. Nat Commun (2021)[9]
PGS000945	Tanigawa Y et al. PLoS Genet (2022)[10]
PGS001348	Tanigawa Y et al. PLoS Genet (2022)[10]
PGS001349	Tanigawa Y et al. PLoS Genet (2022)[10]
PGS001775	Ebenau JL et al. Alzheimers Dement (Amst) (2021)[11]
PGS001828	Privé F et al. Am J Hum Genet (2022)[12]
PGS002249	Lourida I et al. JAMA (2019)[13]
PGS002280	Bellenguez C et al. Nat Genet (2022)[14]
PGS002289	Zimmerman SC et al. JAMA Netw Open (2022)[15]
PGS002731	Xicota L et al. Neurology (2022)[16]
PGS002753	Mars N et al. Am J Hum Genet (2022)(Mars et al., 2022)
PGS003440	Petrican R et al. Sci Rep (2023)[18]
PGS003441	Petrican R et al. Sci Rep (2023)[18]
PGS003574	Mukadam N et al. PLoS One (2022)[19]
PGS003953	Sofer T et al. Alzheimers Res Ther (2023)[20]
PGS003954	Sofer T et al. Alzheimers Res Ther (2023)[20]
PGS003955	Sofer T et al. Alzheimers Res Ther (2023)[20]
PGS003956	Sofer T et al. Alzheimers Res Ther (2023)[20]
PGS003957	Sofer T et al. Alzheimers Res Ther (2023)[20]
PGS003958	Sofer T et al. Alzheimers Res Ther (2023)[20]
PGS003992	Monti M R et al. medRxiv (2023)[21]
PGS004008	Monti M R et al. medRxiv (2023)[21]
PGS004034	Monti M R et al. medRxiv (2023)[21]
PGS004062	Monti M R et al. medRxiv (2023)[21]
PGS004092	Monti M R et al. medRxiv (2023)[21]
PGS004116	Monti M R et al. medRxiv (2023)[21]
PGS004146	Monti M R et al. medRxiv (2023)[21]
PGS004227	Green RE et al. Alzheimers Res Ther (2023)[22]
PGS004228	Green RE et al. Alzheimers Res Ther (2023)[22]
PGS004229	Green RE et al. Alzheimers Res Ther (2023)[22]
PGS004588	Jung SH et al. JAMA Netw Open (2022)[23]
PGS004589	Jung SH et al. JAMA Netw Open (2022)[23]
PGS004590	Lake J et al. Mol Psychiatry (2023)[24]
PGS004600	Tomassen J et al. BMC Neurol (2022)[25]

References

[1] Chouraki V, Reitz C, Maury F, Bis JC, Bellenguez C, Yu L, et al. Evaluation of a Genetic Risk Score to Improve Risk Prediction for Alzheimer's Disease. J Alzheimers Dis 2016;53:921–32. https://doi.org/10.3233/JAD-150749.

[2] Desikan RS, Fan CC, Wang Y, Schork AJ, Cabral HJ, Cupples LA, et al. Genetic assessment of age-associated Alzheimer disease risk: Development and validation of a polygenic hazard score. PLoS Med 2017;14:e1002258. https://doi.org/10.1371/journal.pmed.1002258.

[3] Tosto G, Bird TD, Tsuang D, Bennett DA, Boeve BF, Cruchaga C, et al. Polygenic risk scores in familial Alzheimer disease. Neurology 2017;88:1180–6. https://doi.org/10.1212/WNL.0000000000003734.

[4] Zhang Q, Sidorenko J, Couvy-Duchesne B, Marioni RE, Wright MJ, Goate AM, et al. Risk prediction of late-onset Alzheimer's disease implies an oligogenic architecture. Nat Commun 2020;11:4799. https://doi.org/10.1038/s41467-020-18534-1.

[5] Zhou X, Chen Y, Ip FCF, Lai NCH, Li YYT, Jiang Y, et al. Genetic and polygenic risk score analysis for Alzheimer's disease in the Chinese population. Alzheimers Dement (Amst) 2020;12:e12074. https://doi.org/10.1002/dad2.12074.

[6] Najar J, van der Lee SJ, Joas E, Wetterberg H, Hardy J, Guerreiro R, et al. Polygenic risk scores for Alzheimer's disease are related to dementia risk in APOE ε4 negatives. Alzheimers Dement (Amst) 2021;13:e12142. https://doi.org/10.1002/dad2.12142.

[7] van der Lee SJ, Wolters FJ, Ikram MK, Hofman A, Ikram MA, Amin N, et al. The effect of APOE and other common genetic variants on the onset of Alzheimer's disease and dementia: a community-based cohort study. Lancet Neurol 2018;17:434–44. https://doi.org/10.1016/S1474-4422(18)30053-X.

[8] Leonenko G, Sims R, Shoai M, Frizzati A, Bossù P, Spalletta G, et al. Polygenic risk and hazard scores for Alzheimer's disease prediction. Ann Clin Transl Neurol 2019;6:456–65. https://doi.org/10.1002/acn3.716.

[9] de Rojas I, Moreno-Grau S, Tesi N, Grenier-Boley B, Andrade V, Jansen IE, et al. Common variants in Alzheimer's disease and risk stratification by polygenic risk scores. Nat Commun 2021;12:3417. https://doi.org/10.1038/s41467-021-22491-8.

[10] Tanigawa Y, Qian J, Venkataraman G, Justesen JM, Li R, Tibshirani R, et al. Significant sparse polygenic risk scores across 813 traits in UK Biobank. PLoS Genet 2022;18:e1010105. https://doi.org/10.1371/journal.pgen.1010105.

[11] Ebenau JL, van der Lee SJ, Hulsman M, Tesi N, Jansen IE, Verberk IMW, et al. Risk of dementia in APOE ε4 carriers is mitigated by a polygenic risk score. Alzheimers Dement (Amst) 2021;13:e12229. https://doi.org/10.1002/dad2.12229.

[12] Privé F, Aschard H, Carmi S, Folkersen L, Hoggart C, O'Reilly PF, et al. Portability of 245 polygenic scores when derived from the UK Biobank and applied to 9 ancestry groups from the same cohort. Am J Hum Genet 2022;109:12–23. https://doi.org/10.1016/j.ajhg.2021.11.008.

[13] Lourida I, Hannon E, Littlejohns TJ, Langa KM, Hyppönen E, Kuzma E, et al. Association of Lifestyle and Genetic Risk With Incidence of Dementia. JAMA 2019;322:430–7. https://doi.org/10.1001/jama.2019.9879.

[14] Bellenguez C, Küçükali F, Jansen IE, Kleineidam L, Moreno-Grau S, Amin N, et al. New insights into the genetic etiology of Alzheimer's disease and related dementias. Nat Genet 2022;54:412–36. https://doi.org/10.1038/s41588-022-01024-z.

[15] Zimmerman SC, Brenowitz WD, Calmasini C, Ackley SF, Graff RE, Asiimwe SB, et al. Association of Genetic Variants Linked to Late-Onset Alzheimer Disease With Cognitive Test Performance by Midlife. JAMA Netw Open 2022;5:e225491. https://doi.org/10.1001/jamanetworkopen.2022.5491.

[16] Xicota L, Gyorgy B, Grenier-Boley B, Lecoeur A, Fontaine G, Danjou F, et al. Association of APOE-Independent Alzheimer Disease Polygenic Risk Score With Brain Amyloid Deposition in Asymptomatic Older Adults. Neurology 2022;99:e462–75. https://doi.org/10.1212/WNL.0000000000200544.

[17] Mars N, Lindbohm J V, Della Briotta Parolo P, Widén E, Kaprio J, Palotie A, et al. Systematic comparison of family history and polygenic risk across 24 common diseases. Am J Hum Genet 2022;109:2152–62. https://doi.org/10.1016/j.ajhg.2022.10.009.

[18] Petrican R, Paine AL, Escott-Price V, Shelton KH. Overlapping brain correlates of superior cognition among children at genetic risk for Alzheimer's disease and/or major depressive disorder. Sci Rep 2023;13:984. https://doi.org/10.1038/s41598-023-28057-6.

[19] Mukadam N, Giannakopoulou O, Bass N, Kuchenbaecker K, McQuillin A. Genetic risk scores and dementia risk across different ethnic groups in UK Biobank. PLoS One 2022;17:e0277378. https://doi.org/10.1371/journal.pone.0277378.

[20] Sofer T, Kurniansyah N, Granot-Hershkovitz E, Goodman MO, Tarraf W, Broce I, et al. A polygenic risk score for Alzheimer's disease constructed using APOE-region variants has stronger association than APOE alleles with mild cognitive impairment in Hispanic/Latino adults in the U.S. Alzheimers Res Ther 2023;15:146. https://doi.org/10.1186/s13195-023-01298-3.

[21] Monti R, Eick L, Hudjashov G, Läll K, Kanoni S, Wolford BN, et al. Evaluation of polygenic scoring methods in five biobanks reveals greater variability between biobanks than between methods and highlights benefits of ensemble learning. MedRxiv 2023;3:2023.11.20.23298215. https://doi.org/10.1101/2023.11.20.23298215.

[22] Green RE, Lord J, Scelsi MA, Xu J, Wong A, Naomi-James S, et al. Investigating associations between blood metabolites, later life brain imaging measures, and genetic risk for Alzheimer's disease. Alzheimers Res Ther 2023;15:38. https://doi.org/10.1186/s13195-023-01184-y.

[23] Jung S-H, Kim H-R, Chun MY, Jang H, Cho M, Kim B, et al. Transferability of Alzheimer Disease Polygenic Risk Score Across Populations and Its Association With Alzheimer Disease-Related Phenotypes. JAMA Netw Open 2022;5:e2247162. https://doi.org/10.1001/jamanetworkopen.2022.47162.

[24] Lake J, Warly Solsberg C, Kim JJ, Acosta-Uribe J, Makarious MB, Li Z, et al. Multi-ancestry meta-analysis and fine-mapping in Alzheimer's disease. Mol Psychiatry 2023;28:3121–32. https://doi.org/10.1038/s41380-023-02089-w.

[25] Tomassen J, den Braber A, van der Lee SJ, Reus LM, Konijnenberg E, Carter SF, et al. Amyloid-β and APOE genotype predict memory decline in cognitively unimpaired older individuals independently of Alzheimer's disease polygenic risk score. BMC Neurol 2022;22:484. https://doi.org/10.1186/s12883-022-02925-6.
